# Supplementary material for: The DEG/ENaC cation channel protein UNC-8 drives activity-dependent synapse removal in remodeling GABAergic neurons
Source: eLife. 2016 Jul 12;5:e14599. doi: 10.7554/eLife.14599 (PMC4980115; doi:10.7554/eLife.14599)
Supplement: Supplemental file 1. — DOI: http://dx.doi.org/10.7554/eLife.14599.019 [file elife-14599-fig1.docx]

**Supplemental File 1A. *C. elegans* strains used in this study.**

| **Strain Name** | | **Strain Description** |
| --- | --- | --- |
| NC2320 | | *wdIs74 [pttr-39::mCherry] V; otEx2876[punc-8::GFP; elt-2::GFP]* |
| NC2321 | | *unc-55(e1170) I; wdIs74 V; otEx2876[punc-8::GFP; elt-2::GFP]* |
| NC2585 | | *wyIs202[pflp-13::GFP::RAB-3; pflp-13::mCherry]* X |
| NC2480 | | *unc-8(tm5052)* IV; *wyIs202* X |
| NC2861 | | *juIs137[pflp-13::SNB-1::GFP]* II |
| NC2936 | | *unc-8(tm5052)* IV; *juIs137* II |
| NC2994 | | *unc-8 tm5052 IV*; wdEx960[*punc-25::UNC-8::GFP*] |
| CZ333 | | *juIs1[punc-25::SNB-1::GFP; lin-15+]* IV |
| CB1170 | | *unc-55(e1170)* I |
| NC1851 | | *unc-55(e1170)* I; *juIs1* IV |
| FX05052 | | *unc-8(tm5052)* IV |
| NC2387 | | *unc-8(tm5052) juIs1* IV |
| NC2388 | | *unc-55(e1170)* I*; unc-8(tm5052) juIs1* IV |
| KP5348 | | *nuIs279[punc-25::UNC-57::GFP;punc-25::mCherry::RAB-3]* |
| NC2984 | | *unc-55(e1170)* I; *nuIs279* |
| NC2870 | | *unc-8(tm5052)* IV; *nuIs279* |
| NC2873 | | *unc-55(e1170)* I; *unc-8(tm5052)* IV; *nuIs279* |
| ZM54 | | *hpIs3[punc-25::SYD-2::GFP; lin-15+]* X |
| NC1849 | | *unc-55(e1170)* I; *hpIs3* X |
| NC2875 | | *unc-8(tm5052)* IV; *hpIs3* X |
| NC2874 | | *unc-55(e1170)* I; *unc-8(tm5052)* IV; *hpIs3* X |
| NC2319 | | *unc-55(e1170)* I; *unc-119(ed3)* III; *juIs1* IV; *wdEx658* [*punc-25::mCherry, unc-119(+)*] |
| NC2601 | | *unc-55(e1170)* I; *unc-119(ed3)* III; *juIs1* IV; *wdIs86*[*pttr-39::unc-8; unc-119+; punc-25::mCherry; pttr-39::unc-8 antisense*] |
| IZ1607 | | *pflp-13::mCherry::RAB-3* |
| NC3063 | | *pflp-13::mCherry::RAB-3 ;unc-55; unc-8 juIs1 IV* |
| NC2894 | | *unc-8 tm5052* IV; *wpIs39* *[punc-47::mCherry]* X; *wdEx944* [UNC-8::GFP fosmid; *punc-25::mCherry::RAB-3; pceh-22::GFP*] |
| NC3010 | | *unc-55 (e1170)* I; *unc-8 (tm5052)* IV; *wdIs90 [punc-25::mCherry::RAB-3; ceh-22::GFP]*; *wdEx962 [UNC-8::GFP fosmid; pmyo-2::mCherry]* |
| NC3064 | | *unc-55*; *unc-8 juIs1*; *wdEx*977[*pttr-39::UNC-8; punc-47::mCherry*] |
| EG5052 | | *oxIs351[punc-47:ChR2::mCherry; lin-15+ LITMUS 38i]* X |
| NC2211 | | *unc-55(e1170)* I; *oxIs351* X |
| NC2857 | | *unc-8(tm5052)* IV; *oxIs351* X |
| NC2807 | | *unc-55(e1170)* I; *unc-8(tm5052)* IV; *oxIs351* X |
| CB55 | | *unc-2 (e55)* X |
| NC2454 | | *unc-2(e55)* X; *juIs1* IV |
| NC2443 | | *unc-55(e1170)* I; *unc-2(e55)* X; *juIs1* IV |
| NC2834 | | *unc-55(e1170)* I; *unc-2(e55)* X; *unc-8(tm5052) juIs1* IV |
| RB1887 | | *tom-1(ok2437)* I |
| NC2616 | | *tom-1(ok2437)* I; *juIs1* IV |
| NC2709 | | *tom-1 (ok2437)* I; *unc-8(tm5052) juIs1* IV |
| NC2893 | | *juIs1* IV; *oxIs351* X |
| NC3065 | | *tax-6 p675 IV; nuIs279* |
| NC3066 | | *unc-55 e1170 I; tax-6 p675 IV; nuIs279* |
| MQD5 | | *hqIs5 [ptax-6::tax-6::GFP]* |
| NC3067 | | *hqIs5; punc-47::mCherry X* |
| NC3068 | | *tax-6 jh107 IV; wyIs202 X* |
| VC990 | | *cnb-1 ok276 V* |
| NC3069 | | *cnb-1 ok276 V; juIs1 IV* |
| NC3070 | | *unc-55 e1170 I; cnb-1 ok276 V; juIs1 IV* |
| NC3071 | | *unc-55 e1170 I; cnb-1 ok276 V; unc-8 tm5052 juIs1 IV* |
| NC3080 | | *ced-4 n1162 III; juIs1 IV* |
| NC3081 | | *ced-4 n1162 III; unc-55 e1170 I; juIs1 IV* |
| NC3082 | | *ced-4 n1162 III; unc-55 e1170 I; unc-8 tm5052 juIs1 IV* |
| NC3171 | | *juIs1; wdEx993[pttr-39::UNC-8cDNA; punc-25::mCherry::RAB-3; pmyo-2::dsRed]* |
| NC3167 | | *wdEx961 [punc-25::mCherry::RAB-3; ceh-22::GFP]; oxIs22 [punc-49::UNC-49::GFP; lin-15+] II* |
| NC3186 | | *unc-8 tm5052 IV; wdEx961; oxIs22* |
| NC3187 | | *unc-55 e1170 I; wdEx961; oxIs22 II* |
| NC3188 | | *unc-55 e1170 I; unc-8 tm5052 IV; wdEx961; oxIs22 II* |
|  |  | |
